# Baltimore Academy of Medicine

**Published:** 1888-01

**Authors:** 


### BALTIMORE ACADEMY OF MEDICINE.

REGULAR MEETING, NOVEMBER 15, 1887.

### TRACHEOTOMY AND INTUBATION.

Dr. John R. Uhler, in connection with the reversal of patients for dangerous chloroformnarcosis, suggested the same procedure in tracheotomy for two reasons: (1) To allow the tongue to fall forward, and (2) to prevent the insufflation of diphtheritic products into the trachea when the trachea is just opened, for at this very period a sucking in is apt to occur. He thought the membrane was much better expelled by this treatment—a thing which was very important for the after recovery. He preferred, when possible, to operate slowly, and use the ordinary tracheotomy tube. Sometimes a half syncope occurred when the trachea was first opened, but artificial respiration, with the head low, soon brought about recovery. A cough was invariably caused by introducing an instrument into the trachea as far as the bifurcation.

Dr. C. C. Bombaugh asked at what angle the head was inclined backward.

Dr. Uhler said the head was almost perpendicularly placed with pillows under the back of the neck; and he thought that this position would prevent pneumonia, due to the inhalation of blood, etc.

Dr. J. Edwin Michael remarked that there was nothing more variable than the amount of hæmorrhage which occurred after tracheotomy. Dr, Uhler's method had a mechanical advantage. The matter of doing the operation slowly was very important, and should always be done when possible, but often haste was imperative. The amount of blood lost did not always depend upon the slowness of the operation. One of the last which he had done was on a child under four years, and the circumstances were very unfavorable. The parents were told that the child would surely die without the operation, and might die with it.

They requested it. He operated slowly. He exposed the trachea well, and saw there was no bleeding and only slight oozing. He introduced the knife below, and cut upwards. He preferred cutting in this direction because there was sometimes an abnormal situation of the innominate artery, which was generally out of the way, but occasionally stood higher. He cut up in this case, and on introducing the knife there was a tremendous gush of venous blood. The circumstances were very terrifying. He thought the child would die on the table, and had almost given up hope. He could see nothing for blood. He introduced his finger and slipped the tracheotomy tube in along the side of his nail, when hæmorrhage ceased at once, and the child went on to recovery. The child at one time showed signs of asphyxia, and on removing the tube a cast of a part of the trachea was found. He was inclined to think that intubation in this case would have been fatal. He could not say what had caused the hæmorrhage. He had introduced a hook into the upper end of the wound and his finger into the lower end, and found the space between clear, and he had made the incision exactly where he had intended, and could not say where the gush of blood came from. He was much surprised at the good result of the operation, for the child was much shocked. In the next case, a child of less than two years, the operation was resorted to as the last resort. The child did not lose ten drops of blood. He could not understand this, as he operated as far as possible exactly as in the first case. The trachea was no larger than a goose quill. The child died in forty-eight hours. There was no hæmorrhage.

Dr. S. C. Chew asked if in the first case related, the fibrinous cast was a primary formation.

Dr. Michael said it was and that both durmg and after the operation there was plenty of it below.

The President, Dr. W. C. Van Bibber, related a case which he had just seen that day. He was called by Dr. Clagett to see a child, four years old, that had been sick for four days. He examined the throat and found a tough, horny membrane on the tonsils and epiglottis. The child was sitting up in bed playing. The respiratory murmur was audible, and the countenance good, but the breath was unfavorable. Dr. Clagett had so well covered the treatment that he had had nothing to do. As it was disinclined to eat he advised sausage—a favorite remedy of his—because he had so often found that children would eat it when they would touch nothing else, and then its juice contained everything nourishing. This was unsuccessful, and the child steadily refused to swallow. The next day it was much worse; respiration 40, pulse weaker. Under those circumstances he suggested intubation. Dr. Donaldson, in introducing the tube, found that the trachea was stuffed full of the membrane, so that in pushing the tube in, it seemed like loading a gun. This operation was given up. Statistics of one operator showed that in 50 out of 136 cases, 12 recovered, and of those who died two were like his case. He thought this was the first time intubation had been attempted in Baltimore.

Dr. S. C. Chew asked if he passed the tube down between the vocal cords. He thought it was scarcely correct to say that this was the first attempt at intubation. Dr. Donaldson had once tried it with a gum catheter in a child. Dr. Chew had use trypsin once, but that was hardly a fair test. Papoid was said to be useful in such cases, but he had had no experience with it.

Dr. J. Edwin Michael said that Dr. J. H. Chambers, of this city, had practiced intubation with O'Dwyer's tube in at least a dozen cases, and a small proportion had recovered. He was probably the only one who had used it here to any extent. Dr. O'Dwyer had reported about 136 cases, and Dr. Waxham, of Chicago, about 138 cases, and other cases had been reported. Intubation had had many followers at first, but he was inclined to prefer tracheotomy. Comparisons between tracheotomy and intubation should be based upon an exact knowledge of the symptoms for which the operation was performed, and all the facts should be considered. Waxham reported 138 operations, but it was not probable that one man had so many cases of diphtheritic stenosis in so short a time, for which tracheotomy would have been performed. His experience in tracheotomy was small, because there was a well-known prejudice against the operation in this city, and it was done much less frequently here in proportion than in other cities. He had six cases, with two recoveries. They were all cases with persistent stenosis, aphonia and cyanosis, and he believed they would have died without the operation, and was sure that the two that recovered were decidedly saved. In one case there were all the symptoms except cyanosis; and in another case, a child of a Polish Jew, who lived in a dirty garret, the symptoms were most severe, except cyanosis. The treatment consisted of the bichloride of mercury and steam inhalations, and when dyspnœa was well marked an emetic was given. The membranes were rejected, and the child got well, much to the surprise of all, which went to show that some cases of diphtheritic stenosis got well without operative treatment. He hesitated to operate because his rule was not to operate until cyanosis was well marked.

Dr. John R. Uhler said that about sixteen years ago he had intubated his own child, and kept the catheter in twelve to fourteen hours at a time. This was not easy to do unless the child was very tractable. It was not successful, as the tube became gummed up. It wore the tube ten months.

In reply to Dr. A. K. Bond,

Dr. Uhler said he passed the tube to the bifurcation of the trachea, and he did it to cause spasmodic cough to expel any membrane which might be below the opening.

### REMOVAL OF THE UTERINE APPENDAGES. *

#### DISCUSSION.

Dr. T. A. Ashby thought that Dr. Neale had given a carefully prepared paper, and one full of statistical information. He could add nothing to the views expressed. He fully concurred with Dr. Neale in regard to the advisability of operating in a certain number of cases where objective symptoms were not present, and mere subjective symptoms were the only guide. Within the past week he had had occasion to do this operation upon a patient in whom there were no objective symptoms whatever. Subjectively it was indicated. It was a

*See *Maryland Medical Journal*, November 19, 26 and December 3, 1887.

history of dysmenorrhœa, hysteria, total inability to do work one week out of every four; in fact it was a life almost wrecked by periodical disturbances. She had been seen by a number of physicians. Dr. Ashby had dilated the cervix with negative results. He persistently refused an operation until he found it absolutely necessary as a means of subduing symptoms which all other means had failed to relieve. She was a domestic, white, 31 years old, and lived entirely on her work, but it was impossible for her to keep a position longer than a few weeks at a time. On November the 10th he removed both ovaries and tubes. On cutting through the abdomen he found the left ovary atrophied, but the right one normal in size and appearance. One tube was enlarged and much inflamed. Both ovaries were bound down by adhesions, which gave much trouble in their enucleation. This encysted condition of the ovaries was, he believed, the cause of the distressing symptoms. This was the 6th day, and the temperature was 102 degrees, and pulse 100. 104 degrees was the highest point reached. Her general condition was good, and the only unpleasant circumstance was some vomiting on the last day. He thought she would recover*

He thought the operation in this case was clearly justified, but that in some cases ovaries were removed when they should have been left alone. We should be conscientious, and he had tried to be, for he had waited over eight months before operating, and should not have done it then if any other method of treatment had promised greater relief than oöphorectomy. He thought his case agreed with Dr. Neal's in that they showed the beneficial results of this operation. He showed the ovaries, and said he would report the further progress of the case at a future meeting.

Dr. B. B. Browne reported two successful cases, one of which had been a confirmed invalid for ten years, spending her life in bed or on the sofa; had had severe dysmenorrhœa and had taken much medicine. He removed the ovaries, and found no disease to account for her condition. She is now well. In another case

---

* This patient has made a complete recovery.

of a colored girl with menorrhagia and a fibroid condition of the uterus, he removed the ovaries, and now she is well. He thought that the galvano cautery would take the place of oöphorectomy, and he had used it in many cases and with some success. The danger from the current was almost nothing.

Dr. H. P. C. Wilson regretted he had not heard Dr. Neale's paper. He had had some experience in this operation. He thought the operation should not be done hastily, but when everything else failed, we should, with the patient's consent, perform the operation, which converted confirmed invalids into useful women. Dr. F. West had done the first operation in this city, but Dr. Wilson had done the first one by opening the abdomen. His case was a woman who had intense sufferings; was an opium eater; had adhesions about the uterus, uterine fibroids, and was almost bloodless from hæmorrhage. He sent her to the seashore, and she returned no better, and the case seemed hopeless. He operated, She recovered entirely and afterwards married. In the second case the woman, 16 years old, had 8, 10 and 12 hystero-epileptic attacks a day, and the uterus was bound down. Local and constitutional treatment proving useless, he finally operated. The family wished to send her to an asylum. The ovaries were normal in size, and the tubes were double their normal size, and attached to each other and very difficult to remove. Four weeks after this operation she walked about. The day for the operation was the day of her menstrual period, and he did not know this until the time for operation. He did not postpone it, and the second day after the operation the menses appeared, with no nervous symptoms. A third case was similar. Dr. Robert T. Wilson, his son, had operated about two weeks ago, under the same circumstances, and the case was doing well. He was a great advocate of this operation when the cases were properly selected. It did much good.

Dr. Ashby said that one of his cases menstruated three days after the operation, with no unpleasant symptoms.

Dr. Bond asked Dr. Wilson if he would select that time for operating.

Dr. Wilson said he would not select it, nor would he postpone the operation because the patient was menstruating. In one case he did not care to postpone because it was growing warm, and the woman was becoming weaker.

Dr. Neale said he had noticed this point in his paper. According to Hegar and others, it was very common to have a discharge twenty-four hours, or even several days after the operation, and such discharge was the rule, and it was not true menstruation, but probably due to a temporary venous congestion subsequent to the ligation of the vessels in the broad ligaments. In all of his cases he had observed this. He had performed unilateral ovariotomy on one woman in whom this bloody discharge occurred on the second day, and had since delivered her of three children at terms of successive pregnancies.

One of his cases of oöphorectomy proved fatal on the fourth day. The incision had been two inches long, and the operation lasted about twenty to thirty minutes. The diagnosis at the *post mortem* was subacute traumatic peritonitis. He asked the society upon what symptons of peritonitis we should make the diagnosis. The temperature was never above 101°, and the pulse was 100 to 120. There was excessive pain which came on immediately after the operation, and vomiting, but she had had the same symptoms at every menstrual period for three years. There was no abdomir al distension. Profs. Miltenberger, Tiffany, and himself, had agreed in thinking it not avisable to open the abdomen. In the *Medical Record*, of November 5th, a writer suggested the use of saline aperients as a propylactic or curative treatment of peritonitis. It had the effect of draining the vascular and lymphatic system.

Dr. Chew thought the use of saline aperients for peritonitis was an old remedy re-introduced. He thought opium should be given to prevent all peristaltic action. He suggested the possibility of a peritonitis from a failure on the part of the funbrated extremity to grasp the ovule which would fall upon the abdominal cavity.

Dr. Bond thought that the purgatives were recommended in the early stages of peritonitis, and ice in the late stage.

Dr. Neale said the ovule could easily drop into the abdominal cavity without causing peritonitis, as cases of abdominal pregnancy are conclusive evidence of the ovule dropping into the abdomen attested.

Dr. H. P. C. Wilson thought we should do nothing to increase the peristaltic action of the bowels. Rest and quiet were necessary, and the last thing to do was to give purgatives. The best treatment was opium, and plenty of it, and minute doses of calomel, often repeated for nausea.

Drs. Van Bibber and Uhler spoke of the use of salines in typhoid and dysentery.

Dr. Chew spoke of the impossibility of diagnosticating peritonitis at its onset.

